# Ground Anthrax Bacillus Refined Isolation (GABRI) method for analyzing environmental samples with low levels of *Bacillus anthracis* contamination

**DOI:** 10.1186/1471-2180-13-167

**Published:** 2013-07-18

**Authors:** Antonio Fasanella, Pietro Di Taranto, Giuliano Garofolo, Valeriana Colao, Leonardo Marino, Domenico Buonavoglia, Carmine Pedarra, Rosanna Adone, Martin Hugh-Jones

**Affiliations:** 1 Istituto Zooprofilattico Sperimentale della Puglia e della Basilicata, Anthrax Reference Institute of Italy, Foggia, Italy; 2 Istituto Zooprofilattico Sperimentale dell’Abruzzo e del Molise “G. Caporale”, Teramo, Italy; 3 Departement of Veterinary Medicine, University of Bari, Bari, Italy; 4 Istituto Superiore di Sanità, Roma, Italy; 5 Department of Environmental Sciences School of the Coast & Environment, Louisiana State University, Baton Rouge, LA 70803-5703, USA

**Keywords:** *Bacillus anthracis*, Contaminated soil, Isolation, Method

## Abstract

**Background:**

In this work are reported the results of a qualitative analytical method capable of detecting *Bacillus anthracis* spores when they are present in very low concentration in the soil. The Ground Anthrax Bacillus Refined Isolation (GABRI) method, assessed in our laboratory, was compared with the classic method. The comparison involved artificially anthrax-contaminated soil samples (500 spores/7.5 grams soil) and naturally contaminated soil samples collected in Bangladesh during a field investigation.

**Results:**

The results indicated that, in contrast to the classic method, the GABRI method was able to detect *B.anthracis* in all contaminated samples. The GABRI method produces a more sensitive measure of anthrax spore presence significantly different from the standard method. In particular, the latter is more sensitive to the presence of normal soil contaminants.

**Conclusion:**

The main feature of the GABRI method is its ability to strongly reduce the presence of the environmental contaminants, which being much more numerous than *B. anthracis* tend to inhibit their germination and growth making it extremely difficult to visualize any colonies. The reduction of the microbial environment also allows one to be able to culture and test a larger quantity of potentially contaminated soil and to isolate *B. anthracis* when the spores are present in very low concentrations in the soil.

## Background

*B. anthracis* is the causative agent of anthrax, a non-contagious infectious disease that primarily affects herbivores. However, all mammals, including humans, can be involved. Though having almost completely disappeared in the industrialized countries, anthrax is an important public health problem in many Asian and African areas [1].

*B. anthracis* is a Gram positive, capsulated, and spore-forming bacterium. The spores are very robust and can survive in a suitable soil for several decades. In the Kruger National Park (Africa) *B. anthracis* spores have been isolated from animal bones estimated to be about 200 years old [2]. The ability of *B. anthracis* spores to survive outside the body is key for the ecology and evolution of this pathogen. Higgins [3], Minett & Dhanda [4], Van Ness & Stein [5] and Van Ness [6] observed that spores survive in soils rich in organic material and calcium and much better in alkaline soil with pH above 6.0 and a temperature of about 15°C.

M. Hugh-Jones (unpublished data) noted that in Texas after heavy rains depressed areas, locally called *‘pot holes’*, accumulate humus and minerals from the surrounding soil. The pot holes were found to have calcium concentrations 2–3 times higher, phosphorus 6–10 times and magnesium 2 times higher than the surrounding ground, and this creates locally favorable conditions to enable a better survival of spores in places with otherwise unfavourable soil, e.g., sandy loams [7]. However the strong hydrophobicity of the surface and the buoyancy of the spores have an important role in the ecology of the bacterium. Van Ness noted that the outbreaks of anthrax develop mainly during the dry months that follow a prolonged period of rain. These climatic aspects and the fact that the spores are characterized by a high floating capacity suggest that water plays an important role in the ecology of the bacterium. Rainwater, having washed away the surrounding ground, tends to collect in the low lying parts favoring the concentration of spores. This increases the probability that a grazing animal will acquire an infective dose of spores. However it takes time and special natural events to create sites of concentrations of spores which can cause new infections in grazing animals [6].

It is very easy to isolate *B. anthracis* from biological samples. It grows very well on sheep blood agar. The colonies are white, slightly opaque, a pasty consistency, non-haemolytic and margins slightly indented give the typical appearance to “*caput medusae*”.

However the isolation from the soil is much more difficult than textbooks recount due to the presence of telluric contaminants such as yeasts and bacteria, especially spore-formers, closely related to *B. anthracis,* such as *B. thuringiensis, B. cereus, B. mycoides*[8]. The conflicting presence of contaminating bacteria makes it necessary to heat treat a sample to reduce the vegetative forms of this microbial load [9]. However, heat treatment is ineffective against spores closely related to *B. anthracis*, and this necessitates the use of selective medium [10]. Dragon and Rennie (2001) have shown that a selective culture medium is crucial when isolating *B. anthracis* from environmental samples. The “PLET medium” (Polymyxin, lysozyme, EDTA, thallium acetate) and subsequently the Anthracis Chromogenic Agar (CHRA) [10] can be considered semi-selective media that are able to inhibit the growth of several bacteria and encourage those bacilli belonging to the Cereus group.

While the cultivation and the direct isolation of the bacterium from environmental samples can be difficult and time-consuming compared to molecular methods, e.g. PCR, it is still considered the most sensitive method for the detection of *B. anthracis* in environmental samples [11]. In fact, the biomolecular methods based on the amplification of DNA extracted directly from the environmental sample are not very sensitive. It is known that the spores release their DNA with much difficulty and, furthermore, the examined sample may contain chemicals or organic substances that might interfere with the processes of amplification [12]. Finally, the sensitivity of this method is limited by the very small amount of extract which can be examined [11].

In this work we report the results of a qualitative analytical method capable of detecting very low levels of *B. anthracis* environmental contamination. We compare the Ground Anthrax Bacillus Refined Isolation (GABRI) method with the classic method as described in the OIE Terrestrial Manual 2012. The comparison involved artificially anthrax-contaminated soil samples as well as naturally contaminated soil samples collected in farms of Bangladesh that had suffered from confirmed outbreaks of anthrax [13].

## Methods

### Ethics statement

Experiments described in this paper, previously authorized by the Italian Ministry of Health, (DSVET 0003319-P-13/06/2011), have been conducted without using animals.

### Preparation of anthrax spores

The pathogen strain A0843 of *B. anthracis*[14] was seeded on sporulation agar [15] and incubated at 37°C for 24 hours and then at 23°C. Every 10 days it was tested to verify the level of sporulation and when it reached around 90%, the spores were collected in a sterile saline solution. After three washes, the suspension was incubated at 56°C for 20 min to eliminate any residual vegetative forms.

### Preparation of artificially contaminated soil samples

About 500 grams of soil were collected from the public gardens of the city of Foggia (Italy). The sample was tested and found negative for *B. anthracis*. Twelve aliquots of 7.5 grams each were prepared and 500 spores of the *B. anthracis* strain A0843 were added to each aliquot. Six aliquots were examined by the classic method and six aliquots were examined by the GABRI method.

### Naturally contaminated soil samples

In December 2010, eight farms were visited in Bangladesh where there had been confirmed anthrax outbreaks earlier in the year [13]. Soil samples were collected from selected sites on these farms and were sent for analysis to the Reference Anthrax Institute (Foggia, Italy). The list of samples is reported in Table 1.

**Table 1:** Naturally anthrax spore-contaminated soil samples examined by the classic method at three dilution levels and by the GABRI method

**Soil sample** **(Subdistricts of Bangladesh)**	**CFU of *B. anthracis *isolated by classic method**			**CFU of contaminants isolated by classic method**			**CFU of *B. anthracis *and contaminants isolated by GABRI method**	
	**Total of 10 plates**			**Total of 10 plates**			**Total of 10 plates**	
	**Undiluted**	**1:10**	**1:100**	**Undiluted**	**1:10**	**1:100**	**CFU of *B. anthracis***	**CFU of contaminants**
Faridpur	0	4	8	8482	2190	314	394	1622
Sapatul	108	32	0	1380	162	22	256	200
Dhunot	0	0	0	4404	598	60	10	1164
Santhia	120	128	15	4968	826	90	10,000	276
Shahazadpur	0	0	0	1074	100	14	10	280
Ullapara	20	0	0	66	2	0	68	130
Shahazadpur	2	0	0	426	44	2	12	176
Average	35.7	23.4	3.3	2971.4	560.3	71.7	1535.7	549.7

### Classic method for isolation of *B. anthracis*

The method used for the isolation of spores from environmental samples was that described in OIE Terrestrial Manual 2012 [15], with some modifications. For culturing and isolation of *B. anthracis* the TSMP medium was used, consisting in the semi-selective Columbia blood agar added with trimethoprim (16 mg/lt), sulfamethoxazole (80 mg/lt), methanol (5 ml/lt) and polymyxin (300,000 units/lt). Based on our experience, TSMP has the same efficacy of PLET in isolating *B. anthracis* (data not shown). Briefly, to each 7.5 gram aliquot of soil sample were added 22.5 ml of deionized sterile water. After 30 minutes of washing by vortexing, the suspension was incubated at 64°C for 20 min to eliminate any vegetative forms of soil contaminants [16]*.*

From each sample, 10 ml of supernatant were collected and dilutions of 1:10 and 1:100 were made using normal saline solution.

Subsequently, 10 plates of TMSP were seeded with the undiluted suspension (100 μl/plate), 10 plates with the 1:10 dilution and 10 plates with the 1:100 dilution. After 24 and 48 hours of incubation at 37°C, each plate was examined for the presence of suspect colonies of *B. anthracis* and of contaminants. All colonies were counted. *B. anthracis* colonies were identified by Gram staining, colony morphology and anthrax-specific PCRs [17].

### Ground anthrax bacillus refined isolation (GABRI) procedure

To each 7.5 gram aliquot were added 22.5 ml of washing buffer consisting of deionized water containing 0.5% Tween 20. After 30 minutes of washing by vortexing, the suspension was centrifuged at 2000 rpm for 5 min to eliminate gross debris. The supernatant was harvested and then incubated, aerobically, at 64°C for 20 min to eliminate vegetative forms of *B. anthracis.* After incubation, 5 ml of supernatant were added to 5 ml of Tryptose Phosphate Broth containing 125 μg/ml of Fosfomycin. Then, from each sample, 10 plates of TMSP were seeded with 1 ml/plate of the mix and were incubated, aerobically, at 37°C. After 24 and 48 hours of incubation, each plate was examined and the colonies of *B. anthracis* and of contaminants were counted. *B. anthracis* colonies were identified by anthrax-specific PCRs [17].

### Statistical analysis

The comparison between GABRI and standard methods, applied to the soil samples artificially and naturally contaminated, was carried out using the method of Bland and Altman [18].

## Results

The results of GABRI and classic method applied on artificially and naturally anthrax-contaminated soil samples are shown in Tables 1 and 2, respectively. Table 2 reports the results of soil samples, purposefully contaminated with anthrax, evaluated by the classic method at three dilution levels and by the GABRI method. As shown, no anthrax spores were detected in these samples using the classic procedure, even when undiluted suspensions were examined; in contrast, all samples were positive to the GABRI method. With regard to contaminants, the GABRI method revealed a microbial contamination averaging nearly 1.1 colonies per plate, while by using the classic method, the microbial contamination averaged 59.7 colonies per plate in the suspension, 22.2 in the 1:10 dilution and 3.1 in the 1:100 dilution (Table 2).

**Table 2:** Purposefully anthrax spore-contaminated soil samples examined by the classic method at three dilution levels and by the GABRI method

**Soil sample**	**Anthrax spores added to sample**	**CFU of *B. anthracis *isolated by classic method**			**CFU of contaminants isolated by classic method**			**CFU of *B. anthracis *and contaminants isolated by GABRI method**	
		**Total of 10 plates**			**Total of 10 plates**			**Total of 10 plates**	
		**Undiluted**	**1:10**	**1:100**	**Undiluted**	**1:10**	**1:100**	**CFU of *B. anthracis***	**CFU of contaminants**
N.1	520	0	0	0	725	341	124	2	8
N.2	480	0	0	0	714	337	8	2	9
N.3	500	0	0	0	1000	289	54	2	3
N.4	570	0	0	0	225	45	1	6	4
N.5	430	0	0	0	334	29	1	4	15
N.6	500	0	0	0	584	292	2	3	27
Average	500	0	0	0	597	222.2	31.6	3.2	11.0

Table 1 reports the results of naturally contaminated soil samples from Bangladesh, evaluated by both methods. As shown, when these samples were tested by the classic method, spores of *B. anthracis* were detected only in four undiluted samples, in three samples diluted 1:10 and in two samples diluted 1:100. In contrast, all samples resulted positive to GABRI method. This method revealed a microbial contamination averaging nearly 55 colonies per plate, while the classic method averaged 297 colonies per plate in the suspension, 56 in the 1:10 dilution and 7 in the 1:100 dilution (Table 1).

## Discussion

The results confirmed that the GABRI method was more efficient than the classic method in detecting anthrax spores even in samples with low level of *B. anthracis* contamination.

Interesting is the result concerning the reduction of the microbial contaminants: in the anthrax spore contaminated soil samples, the presence of contaminants was significantly reduced when GABRI method was used respect to the classic method (Tables 1 and 2). This result is significant considering that in the GABRI a suspension volume of 1 ml was tested while the classic method a volume of 0.1 ml was examined.

**Figure 1:**
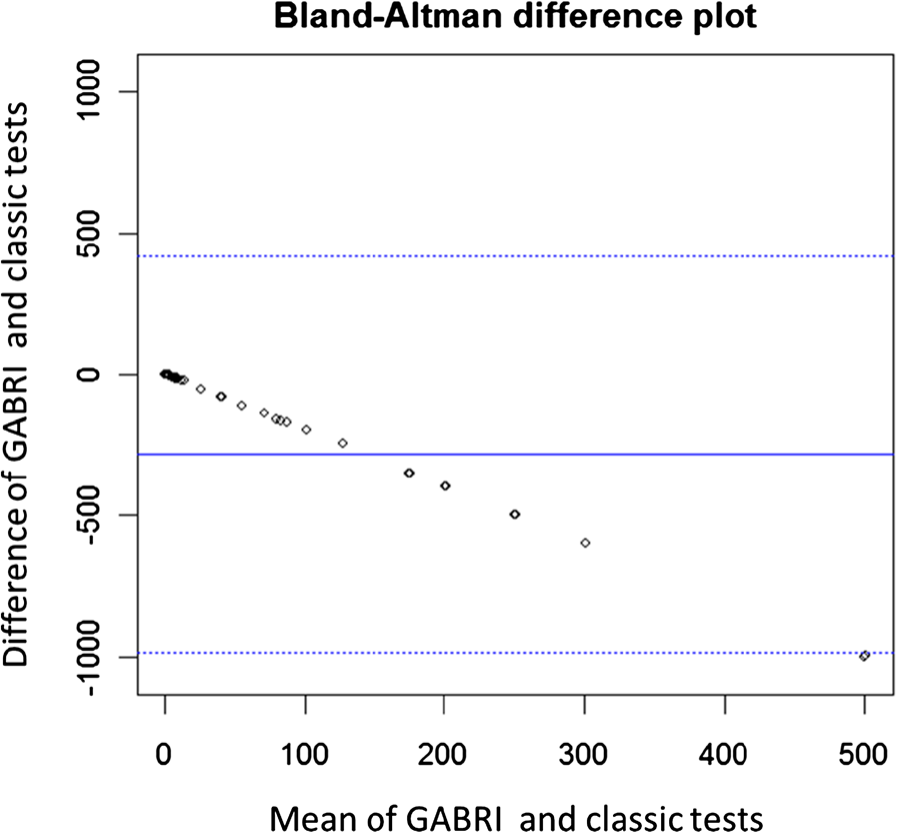
**Bland Altman difference plot indicating agreement between G.A.B.R.I and classic tests.** The mean difference is −282.1 percentage points with 95% confidence interval −377.8 to −186.5 (Standard Deviation = 350.6). The limits of agreement are: Upper agreement limit = 419.1 (95% CI: 253.3 - 584.8 ) and Lower agreement limit = −983.3 (95% CI: -1149.1 - -817.6 ).

The statistical comparison between the two methods was carried out using the method of Bland Altman, through which it was observed that the two methods are not statistically similar (Figure 1). The GABRI method produces a measure of the presence of contaminants significantly different from the classic method. In particular, the classic method is more sensitive to the presence of other *Bacillus* spp., while the GABRI method is more sensitive to the presence of *Bacillus anthracis* compared to the classic method.

To improve the efficiency of classical procedures for detection of anthrax spores in environmental samples, we evaluated a new microbiologic method which in preliminary tests proved to be sensitive and able to distinguish *B. anthracis* from other ubiquitous species.

When environmental samples are tested for the presence of anthrax spores, the main problems are efficiently separating *Bacillus* spores from soil particles and strongly reducing the presence of contaminants which being much more numerous than *B. anthracis,* tend to either inhibit the development of anthrax organisms or just make it extremely difficult to accurately read the cultured result.

The contamination with vegetative cells is not a problem, since they can be easily eliminated by treating samples at temperatures that are lethal for vegetative microbes but not for spores. It has been demonstrated that *Bacillus* spores are hydrophobic and that they adhere to solid matrices especially by mean of hydrophobic interactions [19].

In the GABRI method soil samples were washed for a long time (30 min) with a wash buffer containing 0.5% of Tween 20. As previously demonstrated, in fact, the non-ionic detergents, such as Triton or Tween, allow the separation of spores from soil particles by disrupting hydrophobic interactions with solid matrices [9]. After washing with Tween 20, anthrax spores were recovered from supernatant by centrifugation.

To reduce the presence of contaminants, we treated soil samples with fosfomycin. To evaluate the environmental behavior of *B. anthracis*, Schuch and Fischetti investigated on the role of bacteriophages on bacterial adaptive behavior and niche expansion. In their study, the phage proteins encoding for fosfomycin resistance were specifically described in the spore surface structure of *B. anthracis.* Genes encoding surface proteins and antibiotic resistance may not be virulence factors in the classic sense but can help *B. anthracis* better survive within the highly competitive soil environment [20].

Based on these findings, in the GABRI method supernatants of soil samples, containing anthrax spores, were incubated with fosfomycin (50 μg/μl ) prior to being plated onto selective medium. The results indicated that the treatment with fosfomycin strongly reduced microbial contaminants: the number of colonies of contaminants isolated in plates after GABRI method was significantly inferior to that obtained with classic method (Tables 1 and 2).

The reduced impact of the microbial environment allows the sowing of a larger quantity of a suspension and the isolation of anthrax organisms when they are present in very low concentrations in the soil. *B. anthracis* was isolated from 100% of artificially or naturally contaminated soil samples tested by the GABRI method; in contrast, 43% and 100% of naturally and artificially-contaminated samples, respectively, gave negative results when evaluated by the classic method.

In the classic method usually some 100 μl of the suspension is sown as is and reading these plates can be very difficult. In fact, in the absence of inhibiting actions, the microbial environment is essentially unchanged and the resulting thick carpet of bacteria makes the observation of any *B. anthracis* colonies very difficult, if not impossible. Previous experiments conducted in our laboratory on artificially contaminated soils have confirmed the reduction of the environmental contaminants up to 99% (unpublished data).

## Conclusions

Our results indicate that, due to its ability to strongly reduce contaminants, the GABRI method may be especially suitable for environmental investigations.

Although the GABRI method makes it possible to isolate *B. anthracis* in environmental samples at very low levels of contamination, it should be overemphasized that the most important part of the entire process is the collecting phase. An essential aspect is the collaboration with the farmers because they can give useful, sometimes very accurate information on the actual places where the animals were slaughtered or buried. Moreover, for the pastures considered “infected”, the period of the year when to optimally collect the samples is very important. In regard to historic retrospective investigations we generally recommend that the soil sampling is done in the fall or winter as the pasture grass is short and therefore one can make a better assessment of the orography of the investigated site. The weather conditions are important too. If the soil sampling is done immediately after rain, one has the possibility of taking samples of mud puddles that can appear on an otherwise anonymous slope; these “puddles” can mark the site(s) of cattle graves whose exact location is long forgotten. This system was adopted in Tuscany (Italy) on pastures where years before there had been outbreaks of anthrax in farm cattle. It is necessary to analyze the sample three or four times before declaring it negative.

## Authors’ contributions

AF: Designed, carried out and evaluated all the experimental studies conducted in ABL3 facilities. PDT: collaborated to the experimental studies conducted in ABL3 facilities; GG: carried out molecular assays to identify *B. anthracis* colonies; VC: carried out statistical analysis; LM: collaborated to the experimental studies conducted in ABL3 facilities; DB: collaborated to the experimental studies conducted in ABL3 facilities; CP: prepared all media for culturing and isolation of *B. anthracis*; RA: revised the experimental design and collaborated on the report of the manuscript; MHJ: revised the experimental design and collaborated on the report of the manuscript. All authors read and approved the final manuscript.
